# Novel externalities

**DOI:** 10.1007/s11127-023-01072-x

**Published:** 2023-06-08

**Authors:** Nick Cowen, Eric Schliesser

**Affiliations:** 1 School of Social and Political Sciences, University of Lincoln, Lincoln, UK; 2 Faculty of Social and Behavioral Sciences, University of Amsterdam, Amsterdam, The Netherlands

**Keywords:** Externality, Lippmann, Ostrom, Public health, Pandemics, Epistemic choice, I18, B10, L33, L38, D70, D80

## Abstract

Novel externalities are social activities for which the emerging cost (or benefit) of the spillover is unknown and must be discovered. Negative novel externalities have regained international salience following the COVID-19 pandemic. Such cases frequently are invoked as evidence of the limits of liberal political economy for dealing with public emergencies. Through a re-reading of classical political economy with the modern state’s confrontation with infectious disease in mind, we defend the comparative efficacy of liberal democracy against authoritarian alternatives for coping with these social problems. Effective responses to novel externalities require producing and updating trustworthy public information and an independent scientific community to validate and interpret it. Those epistemic capacities are prevalent in liberal democratic regimes with multiple sources of political power, an independent civil society, and practices of academic freedom. Our analysis highlights the theoretical value of polycentrism and self-governance beyond their more familiar role, of increasing accountability and competition in the provision of local public goods, towards facilitating effective national policy.

## Introduction

A novel externality involves social activities for which the spillover effects are uncertain and must be discovered. When first recognized, all externalities start out novel, of course. In this paper we are especially interested in novel externalities that are taken to threaten public safety and public health, and, by definition, involve the efforts of many scientific disciplines to characterize. Often, they generate a challenge of reconciling public safety with individual liberty. For example, Mittiga (2022) argues that the COVID-19 pandemic justified extraordinary restrictions on freedom of association and that societies may soon face a tradeoff between effective climate change mitigation and maintaining the institutions of liberal democracy.

Some critics present liberal democracy as too slow and inadequately coordinated to deal with externalities of uncertain (but theoretically vast) scale and scope (Geuss, 2008; Fukuyama, 2022), by contrast, argues that policy responses to the COVID-19 pandemic illustrate precisely the resilience of liberal democracy and the poor decision-making of centralized authoritarian states. We agree with Fukuyama and believe that insights from liberal political economy can explain why.^1^

Proponents of interventionist or authoritarian responses to threats to public safety generally characterize public emergencies as justifying exceptional powers (cf. Bjørnskov & Voigt, 2022). Instead, we present novel externalities as a common if unpredictable challenge for public institutions.^2^ Our approach highlights the benefit of an institutional framework that is resilient to potential, or conjectured, emergencies, and that allows for accountability (Lazar, 2009). A focus on the epistemic challenge of externalities shows the benefits of both liberal democratic and market processes even if the two processes also exhibit tensions.

Addressing the problems presented by the COVID-19 pandemic, researchers in liberal political economy have challenged the supposed trade-off between protection against infectious disease and economic freedom (Furton, 2023; Geloso et al., 2021; Geloso & Murtazashvili, 2021; Koyama, 2023), shown that political incentives encourage officials to adopt stricter measures than is economically efficient (Allen, 2022; Boettke & Powell, 2021; Hebert & Curry, 2022; Leeson & Thompson, 2023; Murtazashvili and Zhou this volume; cf. Garzarelli et al., 2022), including measures that persist after the danger has passed (Goodman et al., 2021), proposed that spontaneous endogenous citizen responses to novel infections are more effective than typically predicted (Allen et al., 2022; Leeson & Rouanet, 2021), and highlighted how decisions in the private sector can internalise many of the relevant externalities associated with the infection (Albrecht & Rajagopalan, 2023). Paniagua and Rayamajhee challenge the presumption that the global scale of the pandemic means that national and international policy intervention are where any possible solution lies (Paniagua & Rayamajhee, 2022; Rayamajhee and Paniagua 2022). Rather, what appears to be a global externality is instead constituted by a complex of nested externalities that can ultimately be addressed through adapting local policies (Paniagua 2022).

Much of this existing discussion contrasts voluntary action with government intervention that are characterised as substitutes, often highlighting the under-appreciated benefits of spontaneous voluntary action. Scholars have also explored the quality of government decisions. Pennington (2021) suggests that extraordinary interventions to address infectious disease might be justified but that they sit uneasily with liberal principles; so, they should be suspended at the earliest feasible opportunity. Storr et al. (2021) show that policymakers face a knowledge problem when balancing social distancing interventions with economic production: they do not know what goods and services are ‘essential’ to citizens facing their particular circumstances and needs. Koppl (2023) acknowledges that so long as public agencies exist, citizens will inevitably expect state intervention when faced with novel infectious diseases. The key challenge is avoiding an epistemic monopoly whereby actors granted expert status are able to implement policy uncontested (Murphy et al., 2021; cf. Koppl, 2018, 2021). Bylund and Packard (2021) consider Sweden as a special case that avoided lockdowns in favour of less stringent social distancing measures (compared both to other developed countries and its Nordic relatives). They attribute Sweden’s policy divergence to strong constitutional protections for freedom of association and a formal requirement that any interventions that impact individual liberty be based on scientific evidence.

Winsberg, Brennan and Surprenant (2020) argue that states have epistemic duties when making high stakes state decisions (such as violating civil liberties to combat novel externalities). But ought implies can. Their argument presupposes epistemic capacities that, if they wish to meet their obligations, states must develop. Similarly, Paniagua and Rayamajhee (this volume) propose a useful typology of externalities that distinguishes their scale from the difficulty in internalising them. Such a typology requires a mechanism for establishing the scale and the specific activities responsible for the externality which are initially unknown. Our unique contribution explores the *epistemic* benefits of liberal institutions not only for facilitating spontaneous, endogenous response to novel externalities but *also* for guiding national policy. Evidence, both scientific and practical, that is relevant for addressing externalities is not a given but must be generated, inevitably through trial, error and correction based on observation and learning. The availability and quality of this evidence depends critically on the institutions that govern the creation and sharing of data, and free discourse between scientists, policymakers, and citizens. While choices (both by individuals and policymakers) ultimately involve accepting trade-offs based on subjective values, information helps estimate the nature of the trade-offs. In this sense, trustworthy scientific information is useful both for informing government policy and facilitating effective responses to externalities in civil society and for helping individuals, firms and community groups reconcile the safety of their membership and users with their other commitments.

As the previous paragraph hints, rather than treating state and market as alternatives to each other, we view them as mutually enabling (cf. Paniagua and Rayamajhee this volume). We will argue that novel externalities reveal how the state is required as a machinery of record as an enabling structure for the market to play its epistemic and distributional roles; and that individual agency within and outside markets enables the state to be a more apt machinery of record. A machinery of record facilitates effective feedback and learning within decentralised institutions. Thus, we will show that a focus on state articulacy cuts across familiar debates between state and market. In particular we revive an argument we attribute to John Stuart Mill and Harriet Taylor that shows that the division or decentralization of power might counter-intuitively augment the positive powers of an executive. This contributes to the emerging tradition of mainline economics that adopts an appreciative perspective on the knowledge-generating capacity of institutions that allow for extraordinary cooperation despite individual opportunism and ignorance (Boettke, 2012).

This paper is structured as follows. First, we situate the problem of novel externalities in the broader public choice and liberal political economy programs, highlighting how COVID-19 presents a paradigm example of decision-making amid uncertainty and ignorance. Second, we demonstrate the central role of public health at key junctures in the tradition of liberal political economy. We do so by way of a stylized re-reading of the liberal tradition. Then we explain the fraught challenge that sovereigns face when trying to achieve a collective good from a single central position. We apply this challenge to the production of information. We offer a solution that draws on existing accounts of liberal state capacity and Ostromian understandings of polycentrism. Finally, we indicate how our account of state epistemic capability, which we dub ‘state articulacy,’ can explain some of the variations between national responses to COVID-19.

## Situating novel externalities in liberal political economy

**Fig. 1:**
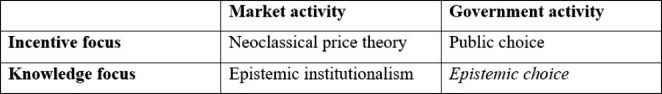
Research Approaches to Liberal Political Economy

The challenge presented by externalities is central to the public choice research program (Marciano, 2013). A core insight is that the mere existence of an externality does not count immediately in favor of state intervention since political activity itself is a source of external costs (Aligică et al., 2019; Buchanan, 2000; Buchanan & Tullock, 1999). At the outset, Buchanan and Stubblebine (1962) acknowledge the challenge of responding to externalities in conditions of uncertainty and ignorance. However, an enduring methodological tension in public choice (Wagner, 2018) led to an initial focus on more measurable challenges, especially decision costs in political processes and transaction costs in civil society. Ostrom (1993, p. 163) highlights this tension in his appraisal of public choice where he identifies the core of public choice research as the application of ‘a nontuistic, self-interested, rational-actor’ model to collective decision processes alongside a periphery that relaxes these assumptions so as to explore how information is created and filtered, as well as how preferences change as part of the process of social interaction (Boettke, 2014) (Fig. 1).

Since Ostrom made that observation, the ‘periphery’ of public choice has been developed. This has included combining epistemological insights from Austrian economics with public choice (Harris et al., 2020). The key premise of Austrian economics is that the knowledge required for the effective coordination of production and consumption is not a given, but must be discovered through experimentation and dissemination (Boettke, 2002; Boettke et al., 2016; Hayek, 1945). People’s capacity to create and disseminate knowledge is dependent on institutions (Palagashvili et al., 2017), namely several property, voluntary exchange and a price system that provides summary information to economic actors about the relative costs of executing their plans. This regime generates new information by allowing actors to make use of their personal, situated, sometimes tacit, knowledge to pursue productive opportunities that others have hitherto not noticed. Successful ventures produce profits for the actor while failures or errors cause them (and their investors) to absorb losses. This account is classically applied to explain the successful production of private goods in market orders. Indeed, Kirzner (2000, p. 77) is sceptical of attempts to apply it in any other setting. Nevertheless, other scholars have identified important implications from the way private markets function on this account for understanding the emergence and maintenance of public governance (Aligică et al., 2019; Leeson & Boettke, 2009). This epistemic focus is particularly important for understanding how states cope with novel threats.

### COVID-19 as a novel externality

We treat a pandemic as an externality because, regardless of its origin, it spreads through human interactions inside and outside market relations. It, thus, differs in character from certain natural disasters (for example, meteorite impacts, tsunamis, and earthquakes). Novelty is a continuous concept rather than a binary distinction. Pandemics – epidemics of an infectious disease with a country-wide even global reach – are part of the human condition thus far. A novel externality need not be novel in kind (or type).

But when a new infection emerges, key features of its characteristics are unknown. COVID-19 provides a paradigm example of a novel externality because an enormous range of information about the costs of hitherto routine individual activity on other people was initially unknown (and some information is, of course, still contested). Moreover, as a pandemic, part of the uncertainty surrounding COVID-19 is how a virus will spread and kill simultaneously in the varied social contexts found globally without a well-established ‘baseline’ case. Hence, while more novel externalities are conceivable (for example, an animal or plant parasite of extra-terrestrial origin with no known antecedents), a new fast-spreading variant of an existing class of viruses lies quite far along the continuum of novelty. Costs are likely to be unpriced and uncontained within established property rights so long as they remain relatively unknown, hence the spillover effects of this behavior constitute externalities. Regarding the virus itself, key unknowns included, but not limited to:

- The infectiousness of the virus and its mechanism(s) of infection.
- The fatality rate for the infection, for whom, and the related co-morbidities.
- The risk of other serious health consequences of infection over time.
- How the virus could mutate over time.
- How protective non-pharmaceutical interventions such as handwashing, ventilation, distancing, and mask-wearing were against infection.

Regarding knowledge about medical responses to the virus, key unknowns included, but not limited to:

- The availability and effectiveness of frontline medicine in treating infection.
- If vaccines could be discovered at all and within a timeframe that could influence the impact of the virus.
- When vaccines could be produced at scale after discovery.
- How effective vaccines would be at protecting against infection and preventing transmission from the infection.
- The scale and severity of adverse side-effects associated with vaccination.
- The availability and effectiveness of protective medical gear to medical practitioners and caregivers.

Many of the possible policy interventions were novel too, and thus also presented great uncertainties including but not limited to:

- How social distancing measures would impact economic activity.
- How social distancing measures would impact long-term social welfare such as the education, socialisation, and mental health of children.
- To what extent businesses and voluntary associations could adapt to social distancing measures.
- The degree of legal enforcement required to achieve a social distancing policy.

Absent information on how these factors would interact with one another, citizens and policymakers had to act with a large degree of conjecture about what the consequences of the spread of the virus and attempts to mitigate it might be, presenting both a risk of policy failure and of unintended consequences. Policymakers and experts also faced challenges of integrating different kinds of scientific expertise from epidemiology and virology to public health, economics, and all kinds of other social sciences in the context of processes that are characterized as ‘fast science’ (Stegenga, 2020). Fast science involves rapidly developing and evolving scientific debates often across disciplines that need to improvise the construction of shared vocabulary and measures in the context of loosening of scientific standards (of peer review and publication) in the service of tackling a novel externality. The choice to wait until better information was available itself presented substantial risks (Norman et al., 2020). When it comes to novel externalities, policymakers do not know which scenario to fear more: the consequences of inaction or the consequences of action amid ignorance. Over the course of the pandemic, many of these unknown factors became progressively known and better understood (Murtazashvili and Zhou This volume).

From the standpoint of epistemic choice, this progressive amelioration of ignorance cannot be treated as a given but rather must be explained through the coordinated and improvised actions of individuals working within institutional frameworks. Under which institutional frameworks have individuals been able to generate the most useful knowledge for dealing with the pandemic? Our contention is that it has been liberal institutions. An appropriate focus on the knowledge problem facing policymakers challenges the notion that authoritarian regimes possess significant advantages for dealing with pandemics, and novel externalities more broadly. In the next session, we offer a stylized re-reading of the classical political economy tradition that shows how liberal thinkers have struggled with this tension between maintaining a general framework of rules and permitting expediency to deal with emergencies.

## The liberal state and public health

Following canonical readings of Hobbes (1651), contemporary political thought usually departs from the presumption that the traditional function of the state is to prevent violent conflict over interests, resources and morality. The state secures people’s safety from the threats of others by establishing and defending borders against foreign powers and policing the domestic population. On this account, the biggest threats to safety arise from rivalries within society. Often represented as a prisoners’ dilemma, social interaction absent a state is intrinsically competitive and can only be ameliorated through the coercion of a third party.

The rediscovered salience of infectious disease has provoked a review of this starting point and highlighted that public safety can require extraordinary collective action not to defend against deliberate harm but also against the unintentional consequences of social interaction.^3^ Rarely noted before the pandemic, the famous frontispiece of Hobbes’ *Leviathan* shows not just a sovereign whose physical body is constituted by the people but also an attractive city with broad avenues, empty of everyone except figures that on inspection are dressed as traditional plague doctors (Falk, 2011). *Leviathan*, on this re-reading, visually presents an outbreak of infectious disease as a paradigmatic instance of necessary state action, which echoes centuries of European practice. Unlike prisoners’ dilemmas where the conflict is over a rival good, such as a subtractable resource, infectious diseases present something more like a stag hunt where effective coordination is to the benefit of all parties but where the cooperation of the other parties nevertheless must be assured. When it comes to initiating coordinated action, the key barriers for public agencies are knowing what the right course of action is, through understanding the nature of an emerging threat, and issuing guidance that actors in civil society can not only follow but can also be confident that others will follow. The response relies relatively less on the threat of coercion (although quarantine rules were familiar to Hobbes), and more on mutual trust and information since effective coordination is more directly to everyone’s benefit (Rayamajhee et al., 2021).

Since pandemics were regular and devastating occurrences in early modern Europe, it is no surprise that liberal thinkers reflected on them. In his *History of Englan*d, Hume (1757 H 16.2 & 64.27) describes frequent pandemics with urban mortality rates of 20%. He notes instances of social distancing at public rallies as early as the sixteenth century (Hume, 1757 H34.18). He also credits the discretionary powers King Charles II assumed after the Great Fire to impose his aesthetic preferences on building ordinances that unintentionally helped London prevent future recurrences of the plague (Hume, 1757, H 64.43.)

Of course, King Charles II’s actions are illiberal. Locke (1690 CH. XIV) addressed the problems of emergency powers in his chapter, “Of Prerogative,” in the Second Treatise. He writes, “many things there are, which the law can by no means provide for; and those must necessarily be left to the discretion of him that has the executive power in his hands, to be ordered by him as the public good and advantage shall require.” (1690 CH. XIV, Sect. 159). Locke goes on to claim that this discretion is in the service of “public good and advantage” in the context of circumstances “that as much as may be, all the members of the society are to be preserved…for the end of government being the preservation of all.” It is notable that Locke puts this not in terms of the survival of society (the effect of, or instituted by, the social contract), but rather in terms of the preservation of “all” the individuals (the “members”) that compose society. On Locke’s view, the prerogative, thus, really is in the service of the preservation of (individual) life rather than, say, the preservation of the state, and so can serve liberal ends. (This makes sense because for Locke, unlike Hobbes, we can survive just fine outside society and the state.)

While Hume rejects the social contract, he builds on these Lockean ideas in his essay “Idea Of a Perfect Commonwealth.” There he argues for institutional structures under the rule of law, and where there is a procedure to grant carefully delimited emergency under specified circumstances and to ensure post facto accountability (cf. Schliesser, 2018; Lazar, 2009). However, neither Locke nor Hume develop an account of novel externalities. Below we will draw on J.S. Mill and H. Taylor, who do go part of the way.

Although theoretical emphasis on public health as a central government competence waned in the second half of the 20th century, perhaps because of fewer direct experiences of fatal infectious diseases, historically it is central to liberal thought (Epstein, 2004; Koyama, 2023). For example, Walter Lippmann (1938) considers a commitment to public health, and especially the treatment and prevention of infectious diseases, central to modern statecraft (De Waal 2020; cf. Cutler et al., 2006). As Adam Smith (1981i) noted, to “prevent” the “spread” of a most loathsome “disease” deserves the “most serious attention of government.” Quoting Smith in *The Good Society* (1938), the book that laid the foundation for the revival of liberalism, including the foundation of the Mont Pelerin Society, after World War II, Lippmann argued, contrary to Herbert Spencer’s doctrine of *laissez faire*, that public health is “both a relief and a remedy.” Lippmann thought it not just a moral duty, but also politically expedient (Schliesser, 2019). Nor did he think of it as an expenditure that must come at the expense of the economy, for a healthier populace also means a more productive economy.^4^ More recently, Rawls argued that “there are matters which concern the interests of everyone and in regard to which distributive effects are immaterial or irrelevant. In these cases the principle of the common interest can be applied…reasonable regulations to maintain public order and security, or efficient measures for public health and safety, promote the common interest in this sense” (Rawls, 1999, p. 83). So, in the liberal tradition broadly conceived, the state must build up expertise and capacity in public health to protect life and promote economic flourishing.

## The executive’s paradox of power

Although Hobbes diagnosed the problem of mis-coordination effectively, his prescription ultimately was flawed. To coordinate effective action to protect the public, an executive must elicit information from her subordinates, decide on a course of action and ensure her will is carried out through commands and direction of resources. A common intuition is that this is best achieved, indeed perhaps only possible at all, if all relevant institutional power is held in the same position with a unitary will behind it. As Tullock (1965) observes, the citizens’ eye view of a government bureaucracy is that of a pyramid with all power and responsibility ultimately residing at the top.

However, even an effective ruler cannot be fully informed about every detail of her realm, nor can she personally ensure all elements of her will are carried out. The idealised role of an organisational pyramid is to delegate these tasks in such a way that the right information, at an appropriate level of granularity, reaches the right rung of the ladder. The sovereign herself only has the cognitive capacity to handle a few specific items of information. She then must state a broad policy that will have to be implemented through commands to subordinates at various levels.

Although this idealised view is not held by contemporary scholars of government bureaucracy, it has often been a point of departure for theorists. Indeed, it remains a central conceit of welfare economics (Adler, 2021; Buchanan, 1959). For example, it was how Woodrow Wilson conceived government and this informed his attempts to develop a centralised federal bureaucracy in the United States (Ostrom, 2008; Wilson, 1908). In the United Kingdom, it is reflected in the increasingly deprecated norms of cabinet collective responsibility for official government policy and individual ministerial responsibility for actions taken within each department (Flinders, 2000; Palmer, 1995). Despite it being implausible that an individual Minister or Secretary of State could really be personally responsible for all key decisions in a government department or policy area, the fiction is retained to create democratic rituals of accountability in parliamentary democracies. Something similar can be said in terms of congressional oversight (McCubbins & Schwartz, 1984).

The paradox we point to is that the very lack of institutional restraints or autonomous actors to check leadership, which is often understood in terms of increase in formal powers, can reduce the effective power of a sovereign to achieve her ends. A curious finding in development economics is that absolute states where the sovereign has few or no constraints are also comparatively weak states (Johnson & Koyama, 2017; Ma & Rubin, 2019). These are states without capacity to collect resources or provide public goods. For example, authoritarian states struggle far more to collect taxes than liberal states. This is not simply because the sovereign must exercise greater coercion for the sake of survival when her rule is in doubt. Even when no serious rivals exist, an absolute state struggles to generate the productive capacities of the society being ruled. Correlatively, capable modern states have emerged in parallel with the growth of civil society, private commerce, administrative capacity and the rule of law (Cox et al., 2019; North, 1990; North et al., 2009).^5^

The kernel of the problem is time-inconsistency. Subjects of a regime can only invest and produce socially valuable resources that can be widely exchanged or shared if they can be confident that they will not be expropriated. Otherwise, out of self-preservation, they must focus on producing for their own private necessities and hiding any surplus from the jealous sovereign. They remain safer by remaining poor. An arbitrary ruler has limited credibility to promise not to expropriate. Hobbes’ expectation was that arbitrary government would be at least as good as any other government because a sovereign’s interests are sufficiently aligned with the majority. This was, in fact, also Hume’s assumption (1739 T3.2.7.1, SBN 534). The problem is the personal interest of a ruler can frequently diverge from that of individuals in civil society. The same powers used to enforce social order can easily be implemented to single out individuals or groups for arbitrary penalty. The occasional emergence of protective states historically happens when they are subject to competition for governance provision (Backhaus & Wagner, 1987; Piano & Salter, 2020).

This is a challenge that crops up repeatedly in any social relationship where one actor functions as a dictator. The readily apparent benefits of being a dictator are undermined by the lack of scope for long-term cooperation. Our observation is that the resources that a dictator wants but cannot produce include data that is useful for guiding policy, including policies that the dictator might otherwise wish to pursue. It is widely established that authoritarian regimes rely on lies and deception to legitimate their rule (Kuran, 1997). For example, dictatorships propagate over-estimates of their country GDPs (Martínez, 2022). In kleptocracies corruption down the chain of command is encouraged in order not just to reward loyalty, but also to maintain leverage over ones cronies (Buckley, 2018). This undermines the impartiality and competence of state bureaucracies.

On our account, not only do authoritarian regimes lie about critical metrics; they also lack the capacity even to produce accurate, trustworthy metrics because neither their bureaucracies nor independent actors in civil society have the security to develop truthful records and share them openly.^6^ This was famously the case for the Soviet Union (Nutter et al., 1962) where official data dramatically overestimated industrial production. Indeed, misplaced faith in data produced by communist dictatorships was sufficiently influential that it impacted mainstream economic thought in the Western world (Levy & Peart, 2011).

How does this relate specifically to bureaucracies and agencies tasked with addressing novel externalities? When taking instructions from a powerful executive, the equivalent of expropriation is loss of employment or reduced prospects of promotion. Officials who have an unclear set of competencies and responsibilities will not take initiative lest they act beyond their powers or get involved in a failed policy for which they might later be blamed. They will wait for explicit instructions from higher up the chain of command so that failure is shared. Because the threat is novel, some degree of failure in retrospect is virtually guaranteed. Critically, officials will avoid conveying accurate information that is unlikely to be well-received by the executive. The paradox is that executives at the top of the bureaucracy may well want intelligent, self-directed subordinates. They may also want to be challenged and to be informed of bad news so they can act to defend the public interest. The problem is they cannot credibly promise not to ‘shoot the messenger’ *ex post*.

## Epistemic institutionalism in public administration

How can a state overcome the paradox of power to produce the essential information for handling infectious disease? Development economists have argued for the centrality of private property, voluntary exchange and enforcement of contracts within a framework of the rule of law for sustained growth and prosperity (Acemoglu et al., 2005; Acemoglu & Johnson, 2005). The Ostroms developed, in parallel, an institutional analysis of public administration to argue for the usefulness of polycentricism (Ostrom, 2010; Ostrom & Ostrom, 2014). They argue regimes constituted by organisations with overlapping jurisdictions and functions, but distinct sources of authority and mechanisms of accountability are more likely to produce the knowledge and incentives necessary for the effective provision of local public goods (Aligica and Tarko 2012, 2013; Sørensen & Ansell 2021).

We argue that as well as local and common goods, in some circumstances, polycentrism is critical to improving the capacity for centralised governments to respond to national crises. The first key contribution is the production, aggregation and dissemination of granular, up-to-date information. This proved to be a critical contribution to dealing with the COVID-19 pandemic, both for national governments getting early-warnings of infections within communities and for being able to track individual cases as well as formulating collective responses. For Lippmann (1922), a core function of the state is as a machinery of record, a collector and disseminator of accurate public data. A lot of our social practices, inside and outside the market, presuppose a social infrastructure in which the machinery of record is reliable, allowing public authorities and private actors to plan their activities. For that to happen, the public must be well-informed, and the only way citizens can possibly be well-informed on complex matters of policy is for state experts to organize and process information.^7^

The germ of Lippmann’s idea is expressed in the closing paragraph of John Stuart Mill’s and Harriet Taylor’s *On Liberty* (1869, Ch. 5), where they claim that “the greatest dissemination of power consistent with efficiency” should be allied with “the greatest possible centralisation of information, and diffusion of it from the centre.” In context, (they invoke the “municipal administration” in “New England”) it is clear that they are responding to Tocqueville’s fears that in order to prevent democratic despotism through local self-government, incompetent art of government is inevitable. Their response to this fear is to make the central bureaucracy a source of epistemic competence, and what we will call a machinery of record, but to attach its expert, civil servants to local government. While we don’t copy their solution, ours is in their spirit.

To be a machinery of record requires state expert bureaucracies that operate by clear and impartial rules. Statistical offices that collect data, and public record keeping that is reliable and accessible to all at minimal cost. This immense and nearly invisible machinery makes possible both political contestation and a great deal of individual and collective decision making. Lippmann calls attention, in particular, to the state’s role in recording births, marriage, death, deeds of ownership, and licensing. We are, of course, not the first to note the importance of record keeping to modern statecraft. In recent decades this insight has been taken up by Foucault (2008) and Scott (1998) and their followers, primarily in terms of making populations legible to the state. We, by contrast, emphasize the state’s capacity to make novel externalities legible to decision-makers and civic society, including by drawing on private expertise and decentralized and/or market discovery procedures. We call this capacity ‘state articulacy.’

While all states collect information, the distinctly liberal state does so in a rule-following fashion and respecting certain limitations. These include the possibility of publicly reconstructing and auditing the construction and sharing of data and so making it contestable. The problem is that building up accurate data collection is challenging without pre-existing capacity and, critically, broad citizen trust in the process. In practice, collection processes that are known to be reliable through use are much more likely to be trusted and trustworthy, and so also have more compliant data sharing. It is, however, impossible to know from theory or formal specification that a data collection process works as it should. Moreover, there is a political economy to data collection when the results determine how resources will be allocated. By contrast, data regularly and consistently gathered by governing units at a smaller scale, and practically used to achieve a variety of local public good needs is likely also to be useful during a national crisis. This can only be relied upon if the system of data collection is subject to feedback as to the success and failure of local interventions.

The current most impressive example of such data collection is the Danish Civil Registration System founded in the 1960s (Pedersen, 2011). This is an administrative register that keeps track of many key details of individuals including those pertinent to uses in public health (Erlangsen & Fedyszyn, 2015) and in epidemiology (Schmidt et al., 2014). Because Denmark’s government has a high degree of transparency *and* accountability and so authority and trust, this administrative register functions smoothly without much social contestation. While we recognize that such a registry has roots in Denmark’s culture of communitarian-organic social democracy with a tendencies toward eugenics (Lucassen, 2010), Denmark’s present public culture and institutions are a robust check on illiberal uses of such a registry, although one should be alert against abuses.

A second contribution is the capacity for liberal states to facilitate informed discussion and debate on novel threats to the society. Berggren and Bjørnskov (2022) show that academic freedom, combined with a protective legal system, is associated with greater levels of productivity, essentially highlighting novel opportunities for entrepreneurs to pursue. Such institutions are also critical for facilitating adaption to new threats both for citizens and policymakers. Evidence for this process can be found in the way universities, private enterprises and research institutes collaborated (and competed) to aggregate, present and interpret data on the COVID-19 pandemic.

Proponents of the necessity of authoritarian responses to pandemic conceptualise this very dissonance of scientific disagreement being played out in public as a limitation on effective government action because it provides cover for the spreading of misinformation. By contrast, liberals acknowledge that no one has a monopoly on scientific truth (Cowen & Trantidis, 2021; Koppl, 2021, 2023; Murphy et al., 2021; Polanyi, 1945, 2000). Rather, science is an ongoing process of competition that involves constantly testing prevailing beliefs against new data and analysis. Especially in the context of novel externalities, it is important not try to impose a hasty consensus, and to allow different disciplines to correct each other (Schliesser & Winsberg, 2020). This is evidenced precisely by policymakers changing their position frequently on key issues such as effectiveness of masks and the risks associated with vaccines. Within a liberal regime, it is acceptable, and necessary, for policymakers to change their minds, but hypocritical and damaging to police the boundaries of opinion that can be expressed.

A third contribution is the capacity of governments to draw on expertise in civil society and the private sector. As Hayek (1945) observes, it is impossible for one actor to know all the available resources, scarcities and needs across a whole society. This is what explains the critical role of decentralised markets with competitive pricing for the coordination of the production of private goods, and the importance of entrepreneurship for generating and dynamically improving public knowledge of available resources and how they can be effectively used. Public officials can never be placed in precisely the same position as entrepreneurs in private markets, where there is a tight link between satisfying ultimate consumer needs and the realisation of profit and loss (Pennington, 2003). Nevertheless, the provision of public goods can be made more effective by bringing accountable decision-making to a more local level. Officials in such a position are more likely to be aware of the immediate needs of their community and the peculiar local resources that might be commissioned. Here, Lippmann (1922) saw value in the circulation of experts between different levels of government, research and even business. For him, a ‘revolving door’ between government and civil society was a strength of a liberal regime rather and not necessarily an avenue for special interests to infiltrate the state. Critically, so long as those responsible for spending public money are locally accountable and transparent (Lazar, 2009), and there is at least the possibility of competition and comparison between providers, then the resources can be effectively deployed whether publicly owned or commissioned or contracted for from the private sector.

Taken as a whole, polycentric institutions split the stylized administrative pyramid that Tullock identifies with bureaucracy into independent units with limited responsibilities. This reduces the need for surveillance and the loss of information that happens as it travels up through fewer layers before it is used (Ostrom & Whitaker, 1973). Nevertheless, these units can exchange information with one another either directly or through state and international agencies or bureaucracies. Because the information is already tested locally, it can be trusted by national governments as generally credible and treated as a plausible official record. In turn, this means that policy decisions taken and announced to the public that are justified by official statistics are more likely to be treated as based on credible information. This can increase personal adherence to governments directives that cannot feasibly be enforced through sanctions.

A possible objection to our account is that, in practice, virtually all liberal democratic governments, regardless of their degree of articulacy, failed to utilise the knowledge made available through these institutions to make effective and proportionate decisions during the pandemic. It could be argued that policymakers were far too quick to compromise on civil liberties (Chenoweth, 2022; Garzarelli et al., 2022) such as freedom of assembly and protest, and stymied genuine public and scientific debate through secretive online censorship (Britschgi 2023). Moreover, instead of drawing and reflecting on local knowledge, policymakers frequently succumbed to group-think especially when introducing lockdowns and other social distancing measure (Chaudhuri, 2022). These practices attenuate the learning processes we affirm since localities did not experiment independently and discouraged open discussion and reporting on key issues. Our response is that despite these actions, some learning took place and, as this pandemic passes into history, more thorough learning from the data collected is feasible. For example, variations in policy approaches have allowed for the development of datasets such as an independent lockdown stringency index (Hale et al., 2021). These datasets, in turn, are allowing scholars to explore the nature of hitherto unmeasured trade-offs between lockdowns, mental health and economic activity (Aknin et al., 2022; Bajra et al., 2023; Cepaluni et al., 2022). There is thus scope to learn from past experiences and errors, and of course to penalise responsible political factions at subsequent elections. By contrast, authoritarian regimes lack much of the credible data and freedom of discussion to permit such learning.

Our assessment is explicitly comparative rather than idealistic (Boettke et al., 2007; Demsetz, 1969). We do not claim that real-world democracies were effective at handling the pandemic compared to an optimal baseline where policymakers are far-sighted and benevolent, only that policymakers working within liberal institutions performed well compared to feasible authoritarian alternatives that have attracted praise. While many restrictions were unjustified (even taken on their own terms), liberal democratic governments have at least been obliged to correct some of their mistakes more quickly and systematically than would have been the case absent the feedback mechanisms we have theorized here. We illustrate this in the final section.

## Liberal institutions and pandemic responses

Can this theoretical notion of state articulacy help explain the differential responses of states to COVID-19? The pandemic revealed some states with initially bad and even disastrous policy responses (China, the United States, the United Kingdom and Italy) and those with rather successful initial responses such as South Korea, Japan, Germany, Taiwan and Czechia (Pancevski and Hinshaw 2020). The explanation for these outcomes cuts across familiar ideological debates about the role of markets and governments. The United States, for example, is the only developed nation without a universal health care system. Hence, some critics argue that “rapaciously profit-driven health care system and an austerity-ravaged state will make this virus harder to manage” (Kapczynski and Gonsalves 2020).

Yet, universal health care was not sufficient to deal effectively with a public health crisis. The United Kingdom has had the National Health Service (NHS) since 1948. Whereas most developed states use a combination of public support, private insurance and co-payments to provide universal healthcare, the NHS is a single-payer system that is free at the point of use (apart from some low flat fees for items like prescriptions). The UK did not perform well against the pandemic either. Just like the United States, the UK was slow to roll out testing and could not get contact tracing off the ground (Barker et al. 2020). The NHS sent countless carers to treat COVID-19 patients without adequate PPE equipment, and its staff were told to keep quiet about it (Campbell 2020). This constitutes many of the same failings of the United States, but playing out within what appears to be a diametrically different health care system.

Why the similar experience? On our account, despite the US and UK having different health care systems, the public health elements both rely on centralized, highly consequential, yet relatively low-capacity, agencies: the Center for Disease Control (CDC) and Food and Drug Administration (FDA) in the United States, and what was called Public Health England (PHE) in the UK, an agency that was subsequently disbanded on arbitrary grounds. In the UK, there were local officials with expertise in contact tracing that were not deployed (Shabi 2020). These central institutions lacked the resources and coordination to predict and respond nimbly to pandemics. Yet they maintained the regulatory clout to prevent other actors from stepping into the breach. They initially struggled to collect and publicise credible data. The CDC, in response to political pressure, conflated viral and antibody tests (Madrigal and Meyer 2020). The resulting public record was so opaque that even well-informed experts did not know how to correct for them. This is an example of the consequences of a relatively hollow rather than articulate state struggling to produce appropriate records and withstand political interference.

This arrangement contrasts with the success of more decentralized yet better coordinated public health regimes elsewhere. Recognizing the threat of COVID-19 early, South Korea’s regulatory agency fast-tracked approvals for privately developed tests (Terhune et al. 2020). They also used smartphone technology to track the infected (Kim 2020). The South Korean government was thus able to utilize private sector capacity and infrastructure for the public good. South Korea’s response could be attributed to its preparedness in response to the previous SARS outbreak (and so it had already developed know how for responding to a kind of novel externality). But Germany, which did not experience a severe infectious respiratory disease outbreak before in living memory, also built-up testing capacity early in the pandemic. This is partly due to its decentralised testing regime that allowed the local state governments to utilize competitively priced private laboratories and experiment with local standards (Loh and Kresge 2020). This could relate to the observation that within neoliberalism, the German so-called Ordoliberal (Ordos) variant has not shared in the mistrust of state capacity characteristic of U.S. conservatism (Foucault, 2008).

The link between liberal institutions and the production of credible data to inform public health is illustrated in the negative by the Chinese response to the crisis. During early stages of the pandemic, local and national officials suppressed honest data collection and sharing. This allowed the outbreak to turn into an epidemic in the first place, leading to many more fatalities. It was resolved only with the most brutal lockdown on mobility and dissent. China’s secrecy made it much more difficult for the global scientific community to accurately understand the spread of infection (Calhoun 2022). Moreover, the Chinese Communist Party evidently coerced World Health Organization officials into publicly praising its failed response as a success while silencing discussion of the much more successful response within China’s independent and partially democratic enclaves (Chan 2020).

From the perspective of epistemic institutionalism, this is not due to specific leadership failure of the Chinese government. Instead, it is the structure of authoritarianism that judges news and data based on its content rather than its integrity. A comparatively weak administrative state colonised by the CCP does not offer space for the formal rationality of independent researchers and administrators to conduct their critical work. An authoritarian government finds it very difficult to acknowledge bad news even when the practical necessity of hearing it is overwhelming. This is how the Chinese ophthalmologist, Li Wenliang, became a martyr of the crisis and why the Chinese government banned foreign press at the outset (Hollingsworth & Xiong, 2021; Yuan, 2022). This projected the illusion of strength but reduced its own ability to respond to events.

## Conclusion

Liberal political economy identifies regime features like localism, the separation of powers, federalism and polycentricity as generally beneficial for dealing with externalities as well as offering opportunities for individuals to engage in collective self-governance. However, this scholarship has not so far identified the benefits of the separation of governmental powers into autonomous units for central government. Indeed, it is possible to imagine that limits and divisions within government must come at a wary executive’s expense. The best that has been said for political leaders so far is that liberal democracy reduces the costs of losing power, a benefit for a political elite but not to their capabilities in their official role (Weingast, 1997). By contrast, since Taylor and Mill, there has been relatively little discussion of how the division or decentralization of power might *augment* the positive powers of an executive.

Novel externalities highlight the limit of what people working from a single central position can achieve when dealing with national public emergencies. To successfully respond to a rapidly spreading infectious disease, many policies must be implemented spontaneously without central direction. We have emphasized that effective national policy relies on the aggregation of accurate information from local areas. To be considered trustworthy, guidance from leadership must be based on data and evidence that is intelligible and interrogable. Such information can only be produced through cooperation among equals, and not the commands of a centralised hierarchy.

## Footnotes

1 In this paper we use ‘liberal’ in the broad sense covering a heterogeneous tradition that presupposes moral equality and formal equality under the law with a wide range of political institutions of accountability as well as an important role of markets in allocating goods. Following Novak (2023), we employ the term ‘liberal political economy’ as a label for an intellectual lens through which social phenomena can be explored based on assumptions of subjective knowledge, analytical egalitarianism, and methodological individualism (cf. Cowen, 2017; Peart & Levy, 2008).

2 To be sure, not all features of novel externalities are unpredictable; often certain pattern types (but not their token instantiations) are quite foreseeable. The type–token distinction is “one between a general sort of thing [type] and its particular concrete instances [tokens]” (Wetzel 2018).

3 Our approach here could be extended to concerns about externalities more generally and especially those focused on emerging environmental crises.

4 We should acknowledge that, although Lippmann was a critic of racial eugenics, his emphasis on public health occasionally aligned with support for a ‘soft’ eugenics that frequently appears in the thought of liberals who departed from laissez-faire in the first half of the 20th century (Cowen, 2018).

5 In this outline, we have simplified the paradox by treating the executive as if he or she stands totally alone. In reality, authoritarian and liberal democratic regimes exist along a continuum (Congleton, 2001) and authoritarian regimes often have independent branches that parallel (and occasionally transform into) the democratic institutions of a constrained executive and legislature (Congleton, 2010). China has systems of checks and balances that constrain local elites (Zhou, 2020). These institutions grant elite constituents some protection from the ruler and can offer the ruler some independent advice and guidance from a relatively narrow set of perspectives. The critical difference between these institutions and established liberal democracies is that they are closed to the majority of the population and lack systems for replacing unpopular elite members in a rule-like fashion (Trantidis, 2017; Weingast, 1997).

6 The high-quality data and bureaucratic performance of a relatively authoritarian city-state like Singapore presents a potential objection to our argument. Singapore is a rare combination of an authoritarian state that is economically free and globally open yet showing few signs of transition towards democracy (Cheang & Choy, 2021; Lawson & Clark, 2010). Its relative success and stability would benefit from further research. One possible hypothesis to explore is that, as a small state surrounded by potential opponents, Singapore faces similar constraints and opportunities faced by the independent principalities that gave rise to cameralist public administration in the 18th and 19th centuries in what eventually became Germany (Backhaus & Wagner, 1987; Salter, 2016), where elite survival happened to be closely aligned with many of the interests of the general population.

7 In the age of cheap computer power and powerful data collection, private institutions and individuals are capable of organizing and disseminating complex and large amounts of data. But in general, they are not capable of coordinating public policy authoritatively.

## References

[CR1] Acemoglu D, Johnson S (2005). Unbundling institutions. Journal of Political Economy.

[CR2] Acemoglu, D., Johnson, S., & Robinson, J. A. (2005). Institutions as a fundamental cause of long-run growth. In *Handbook of Economic Growth* (Vol. 1, pp. 385–472). Elsevier. http://linkinghub.elsevier.com/retrieve/pii/S1574068405010063. Accessed 3 April 2015

[CR3] Adler, M. D. (2021). The social welfare unction: A new tool for regulatory policy analysis. In S. Grundmann, & P. Hacker (Eds.), *Theories of choice: The Social Science and the law of decision making* (pp. 155–178). Oxford University Press. 10.1093/oso/9780198863175.003.0009.

[CR4] Aknin LB, Andretti B, Goldszmidt R, Helliwell JF, Petherick A, De Neve JE (2022). Policy stringency and mental health during the COVID-19 pandemic: A longitudinal analysis of data from 15 countries. The Lancet Public Health.

[CR5] Albrecht BC, Rajagopalan S (2023). Inframarginal externalities: COVID-19, vaccines, and universal mandates. Public Choice.

[CR7] Aligică PD, Tarko V (2012). Polycentricity: From Polanyi to Ostrom, and beyond. Governance.

[CR8] Aligică PD, Tarko V (2013). Co-production, polycentricity, and value heterogeneity: The Ostroms’ public choice Institutionalism revisited. American Political Science Review.

[CR6] Aligică PD, Boettke PJ, Tarko V (2019). Public governance and the classical-liberal perspective: Political economy foundations.

[CR9] Allen DW (2022). Covid-19 lockdown cost/benefits: A critical assessment of the literature. International Journal of the Economics of Business.

[CR10] Allen DWE, Berg C, Davidson S, Potts J (2022). On Coase and COVID-19. European Journal of Law and Economics.

[CR11] Backhaus J, Wagner RE (1987). The cameralists: A public choice perspective. Public Choice.

[CR12] Bajra UQ, Aliu F, Aver B, Čadež S (2023). COVID-19 pandemic–related policy stringency and economic decline: Was it really inevitable?. Economic Research-Ekonomska Istraživanja.

[CR13] Barker, A., Parker, G., Hughes, L., Payne, S., Hodgson, C., Foster, P. (2020, April 3). Germany’s virus response shines unforgiving light on Britain. *Financial Times*. London. https://www.ft.com/content/c4155982-3b8b-4a26-887d-169db6fe4244

[CR14] Berggren N, Bjørnskov C (2022). Academic freedom, institutions, and productivity. Southern Economic Journal.

[CR15] Bjørnskov C, Voigt S (2022). This time is different?—on the use of emergency measures during the corona pandemic. European Journal of Law and Economics.

[CR16] Boettke PJ (2002). Information and knowledge: Austrian economics in search of its uniqueness. The Review of Austrian Economics.

[CR17] Boettke PJ (2012). Living economics: Yesterday, today, and tomorrow.

[CR18] Boettke PJ (2014). Entrepreneurship, and the entrepreneurial market process: Israel M. Kirzner and the two levels of analysis in spontaneous order studies. The Review of Austrian Economics.

[CR21] Boettke P, Powell B (2021). The political economy of the COVID-19 pandemic. Southern Economic Journal.

[CR19] Boettke PJ, Coyne CJ, Leeson PT (2007). Saving government failure theory from itself: Recasting political economy from an austrian perspective. Constitutional Political Economy.

[CR20] Boettke, P. J., Tarko, V., & Aligica, P. (2016). Why Hayek matters: The epistemic dimension of comparative institutional analysis. In P. J. Boettke & V. H. Storr (Eds.), *Advances in Austrian Economics* (Vol. 21, pp. 163–185). Emerald Group Publishing Limited. 10.1108/S1529-213420160000021006

[CR22] Britschgi, C. (2023, March 17). Researchers pressured twitter to treat COVID-19 facts as “misinformation.” *Reason.com*. https://reason.com/2023/03/17/researchers-pressured-twitter-to-treat-covid-19-facts-as-misinformation/. Accessed 13 April 2023

[CR23] Buchanan JM (1959). Positive economics, welfare economics, and political economy. The Journal of Law and Economics.

[CR24] Buchanan JM (2000). The limits of liberty: Between anarchy and Leviathan.

[CR25] Buchanan JM, Stubblebine WC (1962). Externality Economica.

[CR26] Buchanan JM, Tullock G (1999). The calculus of consent: Logical foundations of constitutional democracy.

[CR27] Buckley, N. (2018). *Corruption and power in Russia*. Foreign Policy Research Institute. https://policycommons.net/artifacts/1341736/corruption-and-power-in-russia/. Accessed 28 November 2022

[CR28] Bylund PL, Packard MD (2021). Separation of power and expertise: Evidence of the tyranny of experts in Sweden’s COVID-19 responses. Southern Economic Journal.

[CR29] Calhoun, G. (2022, January 17). China’s manipulation of covid data: The Two ‘Smoking Guns.’ *Forbes*. https://www.forbes.com/sites/georgecalhoun/2022/01/17/chinas-manipulation-of-covid-data--the-two-smoking-guns/?sh=5e390e7c32f3. Accessed 12 November 2022

[CR30] Campbell, D. (2020, March 31). NHS staff “gagged” over coronavirus shortages. *The Guardian*. London. https://www.theguardian.com/society/2020/mar/31/nhs-staff-gagged-over-coronavirus-protective-equipment-shortages. Accessed 25 May 2023

[CR31] Cepaluni G, Dorsch MT, Kovarek D (2022). Mobility and policy responses during the COVID-19 pandemic in 2020. International Journal of Public Health.

[CR32] Chan, W. (2020, April 3). The WHO ignores Taiwan. the world pays the price. *The Nation*. https://www.thenation.com/article/world/taiwan-who-coronavirus-china/. Accessed 12 November 2022

[CR33] Chaudhuri A (2022). Nudged into lockdown? Behavioural economics, uncertainty and COVID-19.

[CR34] Cheang B, Choy D (2021). Liberalism unveiled: Forging a new third way in Singapore.

[CR35] Chenoweth E (2022). Can nonviolent resistance survive COVID-19?. Journal of Human Rights.

[CR36] Congleton RD (2001). On the durability of king and council: The continuum between dictatorship and democracy. Constitutional Political Economy.

[CR37] Congleton, R. D. (2010). *Perfecting parliament: Constitutional reform, liberalism, and the rise of western democracy* (1st ed.). Cambridge University Press. 10.1017/CBO9780511779251.

[CR38] Cowen N, Boettke PJ, Coyne CJ, Storr V (2017). Why be robust? The contribution of market process theory to the robust political economy research program. Interdisciplinary studies of the market order: New applications of market process theory.

[CR39] Cowen N (2018). Mill’s radical end of laissez-faire: A review essay of the political economy of progress: John Stuart Mill and modern radicalism. The Review of Austrian Economics.

[CR40] Cowen N, Trantidis A (2021). Soft interventionism: A hayekian alternative to libertarian paternalism. Review of Behavioral Economics.

[CR41] Cox GW, North DC, Weingast BR (2019). The violence trap: A political-economic approach to the problems of development. Journal of Public Finance and Public Choice.

[CR42] Cutler D, Deaton A, Lleras-Muney A (2006). The determinants of mortality. Journal of Economic Perspectives.

[CR43] De Waal, A. (2020, April 3). New pathogen, old politics. *Boston Review*. Boston. https://bostonreview.net/science-nature/alex-de-waal-new-pathogen-old-politics. Accessed 25 May 2023

[CR44] Demsetz H (1969). Information and efficiency: Another viewpoint. The Journal of Law and Economics.

[CR45] Epstein RA (2004). In defense of the “old” public health. Brooklyn Law Review.

[CR46] Erlangsen A, Fedyszyn I (2015). Danish nationwide registers for public health and health-related research. Scandinavian Journal of Public Health.

[CR47] Falk F (2011). Hobbes’ Leviathan und die aus dem blick gefallenen Schnabelmasken. Leviathan.

[CR48] Flinders M (2000). The enduring centrality of individual ministerial responsibility within the british constitution. The Journal of Legislative Studies.

[CR49] Foucault M (2008). The birth of biopolitics: Lectures at the Collège de France, 1978-79.

[CR50] Fukuyama, F. (2022). More proof that this really is the end of history. *The Atlantic*. https://www.theatlantic.com/ideas/archive/2022/10/francis-fukuyama-still-end-history/671761/. Accessed 25 May 2023

[CR51] Furton GL (2023). The pox of politics: Troesken’s tradeoff reexamined. Public Choice.

[CR52] Garzarelli G, Keeton L, Sitoe AA (2022). Rights redistribution and COVID-19 lockdown policy. European Journal of Law and Economics.

[CR54] Geloso, V., & Murtazashvili, I. (2021). Can governments deal with pandemics? *Cosmos + Taxis*, *9*(5–6), 54–63.

[CR53] Geloso V, Hyde K, Murtazashvili I (2021). Pandemics, economic freedom, and institutional trade-offs. European Journal of Law and Economics.

[CR55] Geuss R (2008). Philosophy and real politics.

[CR56] Goodman N, Coyne CJ, Devereaux A (2021). Infectious diseases and government growth. The Independent Review.

[CR57] Hale T, Angrist N, Goldszmidt R, Kira B, Petherick A, Phillips T (2021). A global panel database of pandemic policies (Oxford COVID-19 Government Response Tracker). Nature Human Behaviour.

[CR58] Harris, C., Cai, M., Murtazashvili, I., & Murtazashvili, J. (2020). *The origins and consequences of property rights: Austrian, public choice, and institutional economics perspectives* (1st ed.). Cambridge University Press. 10.1017/9781108979122.

[CR60] Hebert DJ, Curry MD (2022). Optimal lockdowns. Public Choice.

[CR61] Hobbes T (1651). Leviathan: Or the matter, forme and power of a commonwealth ecclasiasticall and civil.

[CR62] Hollingsworth, J., & Xiong, Y. (2021). The truthtellers. *CNN*. https://edition.cnn.com/interactive/2021/02/asia/china-wuhan-covid-truthtellers-intl-hnk-dst/. Accessed 12 November 2022

[CR63] Hume, D. (1739). In A. Merivale (Ed.), *Treatise of human nature*. Hume Texts Online.

[CR64] Hume, D. (1757). *The history of Great Britain (Online., Vols. 1–6* (6 vol.). Hume Texts Online.

[CR65] Johnson ND, Koyama M (2017). States and economic growth: Capacity and constraints. Explorations in Economic History.

[CR66] Kapczynski, A., & Gonsalves, G. (2020, March 13). Alone against the virus. *Boston Review*. Boston, MA. http://bostonreview.net/class-inequality-science-nature/amy-kapczynski-gregg-gonsalves-alone-against-virus. Accessed 25 May 2023

[CR67] Kim, M. S. (2020, March 6). South Korea is watching quarantined citizens with a smartphone app. *MIT Technology Review*. Boston, MA. https://www.technologyreview.com/2020/03/06/905459/coronavirus-south-korea-smartphone-app-quarantine/. Accessed 25 May 2023

[CR68] Kirzner IM (2000). The driving force of the market: Essays in austrian economics.

[CR69] Koppl R (2018). Expert failure.

[CR70] Koppl R (2021). Against expertism. Review of Behavioral Economics.

[CR71] Koppl R (2023). Public health and expert failure. Public Choice.

[CR72] Koyama M (2023). Epidemic disease and the state: Is there a tradeoff between public health and liberty?. Public Choice.

[CR73] Kuran, T. (1997). *Private truths, public lies: the social consequences of preference falsification* (1st Harvard University Press pbk. ed.). Cambridge, Mass: Harvard University Press.

[CR74] Lawson RA, Clark JR (2010). Examining the Hayek–Friedman hypothesis on economic and political freedom. Journal of Economic Behavior & Organization.

[CR75] Lazar NC (2009). States of emergency in liberal democracies.

[CR76] Leeson PT, Boettke PJ (2009). Two-tiered entrepreneurship and economic development. International Review of Law and Economics.

[CR77] Leeson PT, Rouanet L (2021). Externality and COVID-19. Southern Economic Journal.

[CR78] Leeson PT, Thompson HA (2023). Public choice and public health. Public Choice.

[CR79] Levy DM, Peart SJ (2011). Soviet growth and american textbooks: An endogenous past. Journal of Economic Behavior & Organization.

[CR80] Lippmann W (1922). Public opinion.

[CR81] Lippmann W (1938). The good society.

[CR82] Locke J (1690). Second treatise of government.

[CR83] Loh, T., & Kresge, N. (2020, April 2). Private labs helped Germany test 1 Million For Covid-19 virus. *Bloomberg*. https://www.bloomberg.com/news/articles/2020-04-02/private-labs-helped-germany-test-1-million-for-covid-19-virus

[CR84] Lucassen L (2010). A brave new world: The left, social engineering, and eugenics in twentieth-century Europe. International Review of Social History.

[CR85] Ma D, Rubin J (2019). The paradox of power: Principal-agent problems and administrative capacity in imperial China (and other absolutist regimes). Journal of Comparative Economics.

[CR86] Madrigal, A., & Meyer, R. (2020, May 21). ‘How could the CDC make that mistake?’ *The Atlantic*. https://www.theatlantic.com/health/archive/2020/05/cdc-and-states-are-misreporting-covid-19-test-data-pennsylvania-georgia-texas/611935/. Accessed 25 May 2023

[CR87] Marciano A (2013). Why market failures are not a problem: James Buchanan on market imperfections, voluntary cooperation, and externalities. History of Political Economy.

[CR88] Martínez LR (2022). How much should we trust the dictator’s GDP growth estimates?. Journal of Political Economy.

[CR89] McCubbins MD, Schwartz T (1984). Congressional oversight overlooked: Police patrols versus fire alarms. American Journal of Political Science.

[CR90] Mill, J. S. (1869). *On liberty*. London: Longman, Roberts & Green. http://www.bartleby.com/130/. Accessed 13 April 2010

[CR91] Mittiga R (2022). Political legitimacy, authoritarianism, and climate change. American Political Science Review.

[CR92] Murphy J, Devereaux A, Goodman N, Koppl R (2021). Expert failure and pandemics: On adapting to life with pandemics. Cosmos + Taxis.

[CR93] Murtazashvili, I., & Zhou, Y. (This volume). Complex externalities, pandemics, and the enduring insights of the Ostroms’ institutionalism for public health policy. *Public Choice*.

[CR94] Norman J, Bar-Yam Y, Taleb NN (2020). Systemic risk of pandemic via novel pathogens – coronavirus: A note.

[CR95] North DC (1990). Institutions, institutional change, and economic performance.

[CR96] North DC, Wallis JJ, Weingast BR (2009). Violence and social orders: A conceptual framework for interpreting recorded human history.

[CR97] Novak M (2023). Freedom in contention: Social movements and liberal political economy.

[CR98] Nutter GW, Bornstein I, Kaufman A (1962). Growth of industrial production in the Soviet Union.

[CR102] Ostrom V (1993). Epistemic choice and public choice. Public Choice.

[CR103] Ostrom V (2008). The intellectual crisis in american public administration.

[CR99] Ostrom E (2010). Beyond markets and states: Polycentric governance of complex economic systems. American Economic Review.

[CR100] Ostrom E, Ostrom V, Sabetti F, Aligică PD (2014). Choice, rules and collective action: The Ostroms on the study of institutions and governance.

[CR101] Ostrom E, Whitaker G (1973). Does local community control of police make a difference? Some preliminary findings. American Journal of Political Science.

[CR104] Palagashvili L, Piano E, Skarbek D (2017). The decline and rise of institutions: A modern survey of the austrian contribution to the economic analysis of institutions.

[CR105] Palmer MS (1995). Toward an economics of comparative political organization: Examining ministerial responsibility. Journal of Law Economics & Organization.

[CR106] Pancevski, B., & Hinshaw, D. (2020, April 12). Poorer nations in Europe’s east could teach the west a lesson on coronavirus. *The Wall Street Journal*. New York, NY. https://www.wsj.com/articles/poorer-eastern-european-nations-could-teach-the-west-a-lesson-on-coronavirus-11586718779?mod=e2fb&fbclid=IwAR0pT8cC4hKGu9Br-BgM14j8nSMwzzEHSheha5o-FK-CIwzOYKj6y4jDsT0

[CR107] Paniagua P (2022). Elinor Ostrom and public health. Economy and Society.

[CR108] Paniagua P, Rayamajhee V (2022). A polycentric approach for pandemic governance: Nested externalities and co-production challenges. Journal of Institutional Economics.

[CR109] Paniagua, P., & Rayamajhee, V. (this volume). On the nature and structure of externalities. *Public Choice*.

[CR110] Peart, S., & Levy, D. M. (2008). *The street porter and the philosopher conversations on analytical egalitarianism*. Ann Arbor: University of Michigan Press. http://site.ebrary.com/id/10355571. Accessed 5 April 2015

[CR111] Pedersen CB (2011). The danish civil registration system. Scandinavian Journal of Public Health.

[CR112] Pennington M (2003). Hayekian political economy and the limits of deliberative democracy. Political Studies.

[CR113] Pennington M (2021). Hayek on complexity, uncertainty and pandemic response. The Review of Austrian Economics.

[CR114] Piano, E. E., & Salter, A. W. (2020). The fundamental coase of development: Property rights foundations of the effective state. *Journal of Institutional Economics*, 1–16. 10.1017/S1744137420000260.

[CR115] Polanyi M (1945). The planning of science. The Political Quarterly.

[CR116] Polanyi M (2000). The republic of science: Its political and economic theory. Minerva.

[CR117] Rawls J (1999). A theory of justice.

[CR118] Rayamajhee, V., & Paniagua, P. (2022). Coproduction and the crafting of cognitive institutions during the COVID-19 pandemic. *Journal of Institutional Economics*, 1–7. 10.1017/S1744137422000078.

[CR119] Rayamajhee V, Shrestha S, Paniagua P (2021). Governing nested externalities during a pandemic: Social distancing as a coproduction problem. Cosmos + Taxis.

[CR120] Salter AW (2016). Post-cameralist governance: Towards a robust political economy of bureaucracy. Economic Affairs.

[CR121] Schliesser, E. (2018). Hume on affective leadership. In P. A. Reed & R. Vitz (Eds.), *Hume’s moral philosophy and contemporary psychology* (1 [edition]., pp. 311–333). New York: Taylor & Francis.

[CR122] Schliesser E (2019). Walter Lippmann: The prophet of liberalism and the road not taken. Journal of Contextual Economics.

[CR123] Schliesser, E., & Winsberg, E. (2020). Climate and coronavirus: the science is not the same. *New Statesman*. https://www.newstatesman.com/business/economics/2020/03/climate-coronavirus-science-experts-data-sceptics

[CR124] Schmidt M, Pedersen L, Sørensen HT (2014). The danish Civil Registration System as a tool in epidemiology. European Journal of Epidemiology.

[CR125] Scott JC (1998). Seeing like a state: How certain schemes to improve the human condition have failed.

[CR126] Shabi, R. (2020, April 6). UK missed coronavirus contact tracing opportunity, experts say. *The Guardian*. London. https://www.theguardian.com/uk-news/2020/apr/06/uk-missed-coronavirus-contact-tracing-opportunity-experts-say

[CR127] Smith A (1981). An inquiry into the nature and causes of the wealth of nations.

[CR128] Sørensen, E., & Ansell, C. (2021). Towards a Concept of Political Robustness. *Political Studies*, 003232172199997. 10.1177/0032321721999974.

[CR129] Stegenga, J. (2020). Fast science and philosophy of science. *Auxiliary Hypothesis Spotlight*. https://www.thebsps.org/auxhyp/fast-science-stegenga/. Accessed 12 April 2023

[CR130] Storr VH, Haeffele S, Lofthouse JK, Grube LE (2021). Essential or not? Knowledge problems and COVID-19 stay-at-home orders. Southern Economic Journal.

[CR131] Terhune, C., Levine, D., Jin, H., & Lee, J. L. (2020, March 18). How Korea trounced U.S. in race to test people for coronavirus. *Reuters*. London. https://www.reuters.com/article/us-health-coronavirus-testing-specialrep-idUSKBN2153BW. Accessed 25 May 2023

[CR132] Trantidis A (2017). Is government contestability an integral part of the definition of democracy?. Politics.

[CR133] Tullock, G. (1965). *The politics of bureaucracy*. Public Affairs Press. https://books.google.co.uk/books?id=xWRPAAAAMAAJ.

[CR59] von Hayek FA (1945). The use of knowledge in society. American Economic Review.

[CR134] Wagner RE, Boettke PJ, Stein S (2018). Buchanan’s Liberal Theory of Political Economy. Buchanan’s tensions: Reexamining the political economy and philosophy of James M. Buchanan.

[CR135] Weingast BR (1997). The political Foundations of Democracy and the rule of Law. The American Political Science Review.

[CR136] Wetzel, L. (2018). Types and Tokens. In *Stanford Encyclopedia of Philosophy*. Stanford, Calif. https://plato.stanford.edu/archives/fall2018/entries/types-tokens/. Accessed 11 April 2023

[CR137] Wilson W (1908). Constitutional government in the United States.

[CR140] Winsberg, E., Brennan, J., & Surprenant, C. W. (2020). How Government Leaders Violated Their Epistemic Duties during the SARS-CoV-2 Crisis. *Kennedy Institute of Ethics Journal*, *30*(3), 215–242. Accessed 1 June 2023

[CR138] Yuan, S. (2022). China expels foreign journalists as coronavirus deaths climb. *Al Jazeera*. https://www.aljazeera.com/news/2020/2/20/china-expels-foreign-journalists-as-coronavirus-deaths-climb. Accessed 12 November 2022

[CR139] Zhou Y (2020). Horizontal “checks and balances” in the socialist regime: The party chief and mayor template. Journal of Institutional Economics.

